# Exercise Dependence and Body Image Concerns Amongst Group Fitness Instructors: A Self-Determination Theory Approach

**DOI:** 10.3389/fpsyg.2021.816287

**Published:** 2022-01-20

**Authors:** Michael S. Reinboth, Jorunn Sundgot-Borgen, Solfrid Bratland-Sanda

**Affiliations:** ^1^Department of Sports, Physical Education and Outdoor Studies, University of South-Eastern Norway, Kongsberg, Norway; ^2^Department of Sports Medicine, Norwegian School of Sport Sciences, Oslo, Norway

**Keywords:** group fitness instructors, motivational regulations, exercise dependence, physical activity, body image concerns

## Abstract

**Introduction:**

Despite the fact that group fitness instructors serve as significant role models with potentially great impact on class participants' motivation for exercise, they are a very under-researched group. The aim of this study was therefore to examine group fitness instructors' motivational regulations for exercise, and how these motivational regulations can predict symptoms of exercise dependence and body image concerns.

**Methods:**

Group fitness instructors from the largest fitness companies in Norway (*n* = 837, response rate: 57%) completed an online survey with reference to the Situational Motivation Scale (SIMS), the Exercise Dependence Scale (EDS), the Eating Disorders Inventory subscales drive for thinness (EDI-DT) and body dissatisfaction (EDI-BD), and their weekly amount of exercise and instruction.

**Results:**

The instructors scored high on identified regulation and intrinsic regulation for exercise. EDS total score was positively correlated with all SIMS subscales and weekly instruction was positively correlated with Intrinsic regulation. Multiple hierarchical regression analyses found that both self-determined motivational regulations as well as external regulation positively predicted their EDS score. External regulation positively predicted EDI-DT, and EDI-BD.

**Conclusion:**

Group fitness instructors seem highly intrinsically motivated for exercise, which is hypothesized to have a positive impact on group fitness class participants. High self-determined exercise behavior does not seem to buffer against symptoms of exercise dependence within this specific population. There is a need for awareness of group fitness instructors who show high exercise dependency scores due to the link to body image concerns, amotivation and external regulated motivation.

## Introduction

Although there is a consensus that physical activity and exercise are beneficial for both psychological and physical health (Piercy et al., 2018), the relationship between exercise and body image concerns is far more complex (Thogersen-Ntoumani and Ntoumanis, 2007). Often, individuals may become dissatisfied with the shape of their body, and may become involved in exercise to modify their bodily appearance. Being physically active may not always help people feel better about their bodies, but can sometimes exacerbate body image concerns (Panão and Carraça, 2020). One group of people who may be especially concerned with how their bodies appear to others are group fitness instructors (Prichard and Tiggemann, 2005; Thogersen-Ntoumani and Ntoumanis, 2007). The fitness center industry has grown rapidly over the past three decades; it is recognized to be an important arena for public health work (De Lyon et al., 2017), and group fitness instructors have been shown to be important role models with potentially great impact on their class participants' exercise behaviors and attitudes (Carron et al., 1996; Thogersen-Ntoumani and Ntoumanis, 2007). The industry has also shown extensive focus on body weight, shape and appearance in communication with its members (D'Abundo, 2007) and a high prevalence of disordered eating behavior has been reported among group fitness instructors (Hoglund and Normen, 2002; Bratland-Sanda et al., 2015). A qualitative study of female aerobics participants (Markula, 1995), showed that participants reported persistent body image dissatisfaction despite their high levels of physical activity participation.

Despite the potential harmful health implications of problematic exercise (e.g., excessive exercise, compulsive exercise, exercise addiction, and exercise dependence) there is a lack of consensus on the actual conceptualization and assessment of problematic exercise *per se* (Alcaraz-Ibáñez et al., 2020). Recently a generic term—“morbid exercise behavior” (MEB) has been proposed, which refers to the presence of an increasingly uncontrollable exercise—related behavior that, regardless of the effective time spent exercising, involves physical and/or psychological harm (Szabo et al., 2018). Although we agree with the conceptualization of this phrase, in the present paper, we will use the term exercise dependence, both for the sake of simplicity, and to be more closely in accordance with the measurement scale used in this study.

Given the negative attributes associated with body dissatisfaction and exercise dependence, it would be helpful to advance comprehension of the predisposing factors for such dysfunctional cognitions and behavior. Hopefully, better understanding of the factors underlying exercise dependence and body image concerns can result in greater insight into the psychological mechanisms behind them; advanced knowledge may also allow for the application of preventive strategies. The motivations behind exercise have been described specifically as the key antecedents of exercise dependence (Ogles et al., 1995; Rodgers et al., 2001). As motivation determines the initiation, maintenance, and completion of relevant behaviors, analyzing fitness instructors' quality of motivation may be key to further our understanding of dysfunctional exercise cognitions and behaviors among this group.

Self-determination theory (SDT) (Deci and Ryan, 1985; Ryan and Deci, 2000) addresses the degree to which human actions are self-determined; i.e., the degree to which people engage in their actions with a complete feeling of freedom of choice and autonomy. Moreover, SDT suggests that motivation toward any given behavior can be either *amotivated* (i.e., absent from either extrinsic or intrinsic motivation), *externally* (i.e., perceived pressure to exercise to obtain external incentive), *introjected* (i.e., exercise to avoid feelings of guilt, shame and anxiety), *identified* (i.e., valuing the benefits and outcomes of the behavior, even though it might not be particularly pleasant), *integrated* (i.e., behavior is constructed in congruence with the other values and needs that make up one's personality), or *intrinsically* regulated (i.e., exercise due to the inherent fun and enjoyment). These categorizations of motivation represent different degrees of internalization of external values and goals and therefore differ in the degree to which they are self-determined or autonomous (Ryan and Deci, 2000). Self-determined motivational regulations are related to more adaptive outcomes regarding exercise behavior compared to controlling regulations and amotivation (Vallerand, 1997; Teixeira et al., 2012), yet very few of the studies in these comprehensive reviews have focused upon motivational regulation and dysfunctional exercise behavior.

There is limited research examining the relationship between motivation and dysfunctional exercise behavior in light of SDT (Hamer et al., 2002; Edmunds et al., 2006; Fortier and Farrell, 2009; González-Cutre and Sicilia, 2012). Overall, most of these studies show that introjected regulation seems to be the strongest positive predictor of exercise dependence, and thus not fully supporting the affect regulation model (Tomkins, 1995). This model assumes externally regulated behavior to be the strongest predictor of exercise dependence, since the exercise dependent individual performs exercise to avoid negative affect with an externally perceived locus of causality. However, the above-mentioned studies also show that self-determined forms of motivation such as integrated and identified regulations usually have a positive association with exercise dependence in both athletic as well as exercising populations (Hamer et al., 2002; Symons Downs et al., 2013), thus not fully confirming the predictions by SDT. To our knowledge, only one study by Thogersen-Ntoumani and Ntoumanis (2007) has applied an SDT approach to the study of body image concerns in a sample of British aerobics instructors. Their findings showed that introjected regulation emerged as a positive predictor of drive for thinness and body dissatisfaction.

In a study combining both qualitative and quantitative methods, Fortier and Farrell (2009) analyzed perceptions of body-image and self-determination to try to explain exercise dependence. Based on the findings by Davis et al. (1997) they divided participants (using two median splits) into four groups based on how much they exercised (exercise behavior) and their psychological commitment to exercising (compulsive exercise mindset). Findings showed that respondents with higher scores on compulsive exercise mindset scored higher on introjected regulation and self-determined forms of motivation compared to those with lower compulsive exercise scores. Interview findings also indicated the importance of body image concerns in dysfunctional exercise.

Body dissatisfaction and exercise dependence have also been considered as key risk factors for the development of disordered eating behavior and eating disorders (Stice et al., 2011; Bratland-Sanda et al., 2015). A recent meta-analysis by Alcaraz-Ibáñez et al. (2020) found a small to moderate sized positive association between exercise dependence and overall eating disorder (ED) symptoms, dietary restraint and body/eating concerns. Consequently, there is a need for a more thorough examination of exercise dependence and body image concerns in relation to motivational regulation for exercise among group fitness instructors.

## Purposes and Hypotheses of the Study

Based on the SDT framework, the present study had two purposes. First, we wanted to examine how exercise regulations predicted exercise dependence, drive for thinness and body dissatisfaction among group fitness instructors. In line with a previous study (Thogersen-Ntoumani and Ntoumanis, 2007), we controlled for age, BMI and gender. Secondly, based on the findings by Davis et al. (1997) and Fortier and Farrell (2009), this study examined differences in motivational regulations for exercise and body image concerns among group fitness instructors with different levels of exercise behavior (amount of exercise and instruction) and exercise dependence. Examining exercise dependence scores and exercise behavior separately is in line with recent research by Vrabel and Bratland-Sanda (2019) showing that exercise behavior is not necessarily always associated with thoughts and attitudes toward exercise. In view of the above-mentioned research the following hypothesis were made:

1. Both non-self-determined and self-determined regulations are positively associated with exercise dependence. Non-self-determined forms of motivation are positively associated with drive for thinness and body dissatisfaction.
2. Group fitness instructors reporting high levels of exercise dependence will report higher levels of non-self-determined regulations and body image concerns, regardless of the amount of exercise behavior.

## Methods

### Participants

Group fitness instructors from the three largest fitness companies in Norway were invited to participate in this study. The inclusion criterion was teaching a minimum of one class per week during the spring semester of 2009. The exclusion criterion was inability to understand the Norwegian language. Of the 1,473 group fitness instructors contacted, 78 instructors had invalid contact information and thus were unavailable for the study. The instructors received written information about the aim and completion of the study and gave their written consent to participation. The response rate was 57% (685 females, 152 males). The current sample was the same as that gathered by Bratland-Sanda et al. (2015). The study was approved by the regional committee for medical ethics in Southern Norway.

### Procedure

This study was conducted as a cross sectional study using self-report through the online survey system Questback (www.questback.com). The leaders of group training at the fitness centers provided the instructors' e-mail addresses for the research group. We did not have permission to receive personal information such as names and postal addresses, therefore instructors with invalid e-mail addresses were unavailable for participation. The instructors were contacted by e-mail, and received up to two reminders in the case of non-response. After providing informed consent, participants completed the self-report measures *via* an online survey.

### Self-Report Questionnaires

The questionnaire included questions about age, height, body weight, total number of hours of exercise per week (including both occupational exercise such as instruction of group training classes, and leisure-time exercise).

Motivation for physical activity was assessed through the Norwegian version of the Situational Intrinsic Motivation Scale (SIMS) (Guay et al., 2000). SIMS is a 16-item scale in which respondents are asked to respond to the stem “Why are you currently engaged in physical activity?.” Each item was rated on a seven-point Likert scale ranging from 1 (“Corresponds not at all”) to 7 (“Corresponds exactly”). The 16 items are divided into four subscales: Amotivation (e.g., “I do this activity but I am not sure if it is worth it”), External regulation (e.g., “Because I am supposed to do it”), Identified regulation (e.g., “Because I believe this activity is important for me”), and Intrinsic regulation (e.g., “Because I think that this activity is interesting”). Cronbach's alpha was 0.72, 0.66, 0.65 and 0.67 for each subscale.

The Eating Disorders Inventory version 2 (Garner, 1991) subscales drive for thinness (EDI-DT, 7 items) and body dissatisfaction (EDI-BD, 9 items) was used to examine body image concerns. The items are ranged on a six-point Likert scale from “never” to “always” with higher score indicating more drive for thinness and/or body dissatisfaction. Cronbach's alpha for the EDI-DT was 0.71 and 0.85, and EDI-BD was 0.90 and 0.89 for the male and female instructors respectively.

Exercise Dependence Scale (EDS) (Symons Downs et al., 2004) was used to assess symptoms of exercise dependence. This 21-item scale is developed on the basis of proposed criteria for exercise dependence (Hausenblas and Symons Downs, 2002; Symons Downs et al., 2004). The items are rated on a six-point Likert scale ranging from “never” to “always.” Higher score on EDS indicates more symptoms of exercise dependence. Cronbach's alpha for the 21 items was 0.88 and 0.87 for the male and female instructors respectively.

### Statistics

The statistical software IBM SPSS 24 was used to analyse the data. Descriptive variables are shown in mean and standard deviation. Independent t-tests were used to explore statistical differences between male and female respondents. Spearman's rho was used to examine correlations between variables. To answer our first hypothesis, we conducted three multiple hierarchical regression analyses with the following dependent variables: EDS score, EDI-DT and EDI-BD. Independent variables for the three analyses were sex, age, BMI and SIMS subscales. To test our second hypothesis, similar to research by Fortier and Farrell (2009), participants were divided into four groups based on their EDS score and their reported total amount of exercise. By using two median splits, we created the following exercise behavior groups: Group 1 = low EDS score, low exercise amount. Group 2: low EDS score, high exercise amount. Group 3 = high EDS score, low exercise amount. Group 4 = high EDS score, high exercise amount. To examine differences between these four groups, we conducted ANOVA with Tukey's HSD *post hoc* test. Significance level was set to 0.05.

## Results

Table 1 presents means and standard deviations for the study variables. Numerical differences were found for gender. Male group fitness instructors were older, had higher BMI, scored higher on amotivation and lower on identified regulation compared to female instructors. Female instructors scored higher on EDI-DT and EDI-BD than males. The highest scores regarding the different types of motivation were intrinsic regulation (*M* = 6.34) and identified regulation (*M* = 6.52) and the lowest scores in non-self-determined forms of motivation (amotivation and external regulation) (Table 1).

**Table 1:** Descriptive data.

	**Males (*n* = 152)**	**Females (*n* = 685)**	**Total (*****n*** **=** **837)**	
	**Mean (SD)**	**Mean (SD)**	**t**	**Mean (SD)**
Age	37.9 (9.5)	32.8 (8.3)	6.64^***^	33.7 (8.8)
BMI (kg/m^2^)	25.5 (2.7)	22.5 (2.4)	13.62^***^	23.0 (2.7)
SIMS				
Amotivation	1.29 (0.81)	1.15 (0.52)	2.70^**^	1.17 (0.58)
External regulation	2.58 (1.29)	2.39 (1.18)	1.75	2.42 (1.20)
Identified regulation	6.44 (0.55)	6.54 (0.57)	−2.31^*^	6.52 (0.49)
Intrinsic regulation	6.27 (0.63)	6.36 (0.62)	−1.70	6.34 (0.62)
EDS total score	57.02 (12.18)	56.44 (11.81)	0.53	56.55 (11.87)
EDI-DT	1.30 (2.47)	2.97 (4.37)	−4.58^***^	2.67 (4.14)
EDI-BD	2.91 (3.91)	4.84 (6.02)	−3.78^***^	4.49 (5.74)
Exercise amount (h/w)^a^	7.45 (3.75)	7.45 (3.80)	−0.20	7.45 (3.79)

*Values are shown in mean (SD)*.

* *p < 0.05*.

** *p < 0.01*.

*** *p < 0.001*.

*BMI, body mass index; SIMS, situational motivation scale; EDS, Exercise Dependence Scale; EDI-DT, Eating Disorders Inventory subscale Drive for Thinness; EDI-BD, Eating Disorders Inventory subscale Body Dissatisfaction*.

a *Exercise amount includes both leisure-time exercise and occupational exercise (i.e., instruction of group exercise classes)*.

A correlation analysis showed that the coefficients among the SIMS subscales were correlated with each other reflecting the theorized simplex-like pattern (Guay et al., 2000). Moreover, the analysis found positive correlations between total amount of exercise and intrinsic regulation. The EDS total score was moderately positively correlated with all four SIMS subscales (Table 2), and showing a strong correlation with external regulation (*r* = 0.24, *p* < 0.01). External regulation was also positively associated with body image concerns. Total amount of exercise was also moderately correlated with EDS total score.

**Table 2:** Spearman's rho correlations.

	**1**	**2**	**3**	**4**	**5**	**6**	**7**	**8**	**9**	**10**	**11**
	**Sex**	**Age**	**BMI**	**AM**	**EXT**	**ID**	**IR**	**DT**	**BD**	**EDS**	**TE**
1	–										
2	−22^**^	–									
3	0.43^**^	0.13^**^	–								
4	−0.09^*^	0.15^**^	0.11^**^	–							
5	−0.0.6	0.02	0.14^**^	0.35^**^	–						
6	−0.08^*^	−0.02	0.12^**^	−0.07	0.09^**^	–					
7	0.06	−0.02	−0.09^*^	−0.01	−0.04	0.58^**^	–				
8	0.16^**^	0.21^**^	0.08^*^	−0.07^*^	0.20^**^	0.01	0.01	–			
9	0.13^**^	0.18^**^	0.29^**^	0.05	0.16^**^	−0.08^*^	−0.06	0.66^**^	–		
10	−0.02	0.25^**^	−0.03	0.14^**^	0.24^**^	0.11^**^	0.18^**^	0.31^**^	0.21^*^		
11	0.00	−0.13^**^	12^**^	0.03	0.00	0.10^**^	0.18^**^	0.11^**^	0.01	0.34^**^	–

* *p < 0.05*.

** *p < 0.01*.

*** *p < 0.001*.

*AM, amotivation; EXT, external regulation; ID, identified regulation; IM, intrinsic regulation; DT, drive for thinness; BD, body dissatisfaction; EDS, Exercise Dependence Scale total score; TE, total amount of exercise (hours per week)*.

To test our first hypothesis, we carried out multiple hierarchical regression analysis. In line with Thogersen-Ntoumani and Ntoumanis (2007) we examined whether the different motivational regulations could predict exercise dependence, body dissatisfaction and drive for thinness, after controlling for sex, age and BMI. The results showed that sex, age, amotivation, external regulation and intrinsic regulation were uniquely associated with EDS score, explaining 16% of the variance (Table 3). Sex, age, BMI and External Regulation explained 21 and 12% of the variance in EDI-BD and EDI-DT, respectively (Table 3).

**Table 3:** Multiple hierarchical regression analysis predicting EDS total score, body dissatisfaction and drive for thinness from sex, age, BMI and motivational regulations with standardized variables.

	**EDS**		**EDI-BD**		**EDI-DT**	
	*F* = 22.58^***^, Adj. *R*^2^ = 0.16		*F* = 30.40^***^, Adj. *R*^**2**^ = 0.21		***F*** = 17.41^***^, Adj. *R*^**2**^ = 0.12	
**Independent variables**	**B (95% CI)**	**t**	**B (95% CI)**	**t**	**B (95% CI)**	**T**
Sex	−0.10 (−5.34, −0.86)	−2.71^*^	0.28 (3.12, 5.2)	7.88^***^	0.21 (1.44, 3.01)	5.56^***^
Age	−0.27 (−0.47, −0.28)	−7.99^***^	−0.18 (−0.19, −0.01)	−5.54^***^	−0.19 (−0.13, −0.06)	−5.71^***^
BMI	−0.06 (−0.57,0.07)	−1.53	0.40 (−0.20, −0.03)	11.33^***^	0.17 (0.15,0.37)	4.58^***^
Amotivation	0.11 (0.86, 3.99)	3.05^**^	0.02 (−0.53,0.86)	0.46	0.04 (−0.21,0.84)	1.17
External regulation	0.21 (1.41, 2.81)	5.92^***^	0.12 (0.27,0.93)	3.60^***^	0.19 (0.42,0.91)	5.26^***^
Identified regulation	−0.01 (−2.17, 1.78)	−0.19	−0.06 (−1.60,0.21)	−1.51	−0.01 (−0.76,0.61)	−0.23
Intrinsic regulation	0.19 (−2.01, 5.15)	4.67^***^	−0.01 (−0.03,0.15)	−0.36	0.02 (−0.39,0.68)	0.53

* *p < 0.05*.

** *p < 0.01*.

*** *p < 0.001*.

*EDS, Exercise Dependence Scale; EDI, Eating Disorders Inventory; BD, body dissatisfaction; DT, drive for thinness*.

An ANOVA revealed significant differences between the four exercise behavior groups on three of the motivation subscales as well as on body image concerns (Table 4). The fitness instructors who scored above the median on EDS (i.e., Groups 3 and 4), displayed significantly higher levels of external regulation [*F*_(3,787)_ = 6.95, *p* < 0.001, partial η^2^ = 0.03], drive for thinness [*F*_(3,789)_ = 19.68, *p* < 0.001, partial η^2^ = 0.07], and body dissatisfaction [*F*_(3,789)_ = 9.76, *p* < 0.001, partial η^2^ = 0.04] compared to those scoring below the median (i.e., Groups 1 and 2). Regarding intrinsic regulation, Groups 2, 3 and 4 scored significantly higher than Group 1 [*F*_(3,789)_ = 9.76, *p* < 0.001, partial η^2^ = 0.04].

**Table 4:** Means (and SD) for groups of fitness instructors on motivation and body image concerns.

	**Group 1 *n* = 281**	**Group 2 *n* = 118**	**Group 3 *n* = 178**	**Group 4 *n* = 216**
	**Low EDS score/Low exercise amount**	**Low EDS/High exercise amount**	**High EDS score/Low exercise amount**	**High EDS score/High exercise amount**
Variable	M (SD)	M (SD)	M (SD)	M (SD)
Amotivation	1.11 (0.44)	1.17 (0.69)	1.16 (0.45)	1.24 (0.60)
External regulation ^*^	2.28 (1.18)	2.17 (1.09)	2.61 (1.18)	2.63 (1.21)
Identified regulation ^*^	6.46 (0.49)	6.56 (0.49)	6.60 (0.41)	6.56 (0.50)
Intrinsic regulation ^*^	6.20 (0.69)	6.41 (0.58)	6.39 (0.54)	6.48 (0.55)
EDI-DT ^*^	1.80 (3.31)	1.31 (2.47)	3.31 (4.45)	4.12 (5.04)
EDI-BD ^*^	3.98 (5.35)	2.57 (4.10)	5.20 (6.03)	5.76 (6.46)

* *p < 0.05*.

*EDS, exercise dependence scale. Exercise amount: hours/week with both occupational exercise and leisure-time exercise. EDI-DT, Eating Disorders Inventory Subscale Drive for Thinness. EDI-BD, Eating Disorders Inventory Subscale Body Dissatisfaction*.

## Discussion

The overall purpose of the present study was to examine the role of motivational regulations to exercise dependence and body image concerns among group fitness instructors, using the SDT framework. More specifically, we wanted to examine how these exercise regulations were uniquely associated with exercise dependence, drive for thinness and body dissatisfaction. Our main findings were that external regulation was the only motivational regulation that predicted all three dependent variables, and that the fitness instructors with EDS score above the median scored higher on external regulation and body image concern compared to EDS score below the median, regardless of actual exercise amount.

Exercising due to other peoples' expectations and because one feels one must, may be detrimental to one's body image evaluations and dysfunctional exercise behavior. More specifically, the two least self-determined regulations, amotivation and external regulation, were positive predictors of exercise dependence. This finding is in line with the affect regulation model by Tomkins (1995). The implications are that individuals who develop exercise dependence initially view exercise as a method to avoid or reduce negative affect. The experience of needing to reduce negative affect without being able to exercise may then transform this relationship into a dependency. As the suffering from not being able to exercise increases, it may replace and attenuate the original suffering from needing to reduce negative affect.

Interestingly, intrinsic regulation was uniquely associated with exercise dependence among this sample of fitness instructors. Previous research (Hamer et al., 2002; Symons Downs et al., 2013) has shown self-determined forms (identified, integrated and introjected regulation) to predict exercise dependence. At first glance, this positive association between exercise dependence and self-determined forms of exercise motivation, does not seem consistent with the basic assumptions of SDT, which proposes that self-determined forms of regulations should lead to positive consequences (i.e., healthy exercise behavior). A recent study by Sicilia et al. (2018) examined how the role of harmonious and obsessive passion mediated the relationship between self-determined motivation and exercise dependence. According to them, a high degree of exercise behavior internalization does not necessarily determine the quality of the process. In their correlational analysis, intrinsic motivation showed moderate association with both obsessive and harmonious passion. A high degree of internalization of the values of exercise may be associated with harmonious passion if the internalization process is developed completely and harmoniously. However, based on their findings, they argue that it is also possible to find self-determined forms of motivation in exercise and maintain an obsessive passion for this activity. It could be that instructors are initially drawn into the business because they are truly dedicated to exercising and see it as a way to make a living out of something they enjoy. Although we did not measure the motivational climate in this study, the work environment of fitness instructors and the signals transmitted by both colleagues and leaders in the industry can be said to be rather competitive with a focus on comparing each instructors' ability to that of other instructors (Maguire, 2001). As noted by González-Cutre and Sicilia (2012), exercise dependence will more likely emerge in a motivational climate that is ego-involving. It could therefore be that the internalization of exercise behavior in such environments occurs in an obsessive manner. It is also possible that this environment, with a focus on obtaining a slender and athletic figure, combined with having their bodies constantly on display (Prichard and Tiggemann, 2005) may lead to challenges such as body image concerns and exercise dependence. However, as suggested by Thogersen-Ntoumani and Ntoumanis (2007), it could be that in this special sample, high levels of self-determined motivation may not be sufficient to protect against development of such challenges.

Overall, the fitness instructors scored rather high on identified (M = 6.52; SD = 0.49) and intrinsic (M = 6.34; SD = 0.62) and quite low on external (M = 2.42; SD = 1.20) and amotivation (M = 1.17; SD = 0.58) similar to scores among elite athletes (Gillet et al., 2013) and to students (Gao, 2008; Lonsdale et al., 2009; Gao et al., 2011). It could be argued that the very high scores on self-determined motivation indicate a ceiling effect and that this potentially influenced the regression analysis. To avoid such bias, we used standardized variables for the regression analysis. Nevertheless, we cannot automatically transfer these findings to other populations with lower mean scores on self-determined motivational regulation. Moreover, external regulation positively predicted body image concerns. None of the self-determined regulations emerged as predictors of neither drive for thinness nor body dissatisfaction. Although the SIMS scale (Guay et al., 2000) used in this study does not include introjected regulation, these results are in line with findings by Thogersen-Ntoumani and Ntoumanis (2007) who found introjected regulations, but not self-determined regulations, to positively predict both drive for thinness and body dissatisfaction in British aerobics instructors. Thus, this adds to the knowledge about unhealthy associations between external regulation of exercise, such as exercise for appearance and weight-related reasons, and body image concerns.

Despite rather small effect sizes, dividing of the fitness instructors into four groups based on their exercise behavior showed some interesting results. First, it is important that those above median score on EDS scored higher on non-self determined regulation and body image concerns regardless of their reported exercise amount. The findings partially support findings by Fortier and Farrell (2009) and, in our opinion, advance knowledge from other recent findings that call for a distinction between exercise cognitions and actual exercise behavior (Vrabel and Bratland-Sanda, 2019). Further, it appears that the high levels of self-determined regulations reported by these groups, does not seem to buffer against dysfunctional exercise behavior. Future research will need to investigate the relationship between exercise cognitions and actual objective exercise behavior in more detail.

## Strengths and Limitations

The large number of respondents, inclusion of both male and female participants, inclusion of a variety of group fitness instructors and use of standardized and validated instruments strengthen the findings of this study. The findings are, however, limited by a relatively low response rate, as well as self-reported amount of exercise rather than objective measures. Also, this study is cross-sectional, and does not provide any information with regards to the instructors' long term, or possible changes in, motivation or body image concern over time. It could be that instructors with very high levels of body image concerns see the job as a fitness instructor as a good solution to deal with or solve their problems. Moreover, longitudinal research is needed to examine these relationships over time. Furthermore, the influence of the motivational work climate in which the instructors operate, on a daily basis, needs to be examined. The scale measuring motivational regulations (Guay et al., 2000) in this study (which was the only well-validated version in the Norwegian language at the time of data collection), did not include introjected regulation nor integrated regulation and could therefore be affecting the interpretation of the study results. Moreover, the EDI-BD was designed to evaluate body dissatisfaction in women and evaluates dissatisfaction with parts of the female body. An instrument to assess muscularity could therefore be applied in future studies.

## Practical Implications

Based on the findings, there is a need for awareness of group fitness instructors who show high exercise dependency scores due to the links between body image concerns and non-self-determined regulation of exercise motivation. The leaders in the fitness industry thus need to gain and improve their competence in how to identify and manage such challenges among their employees. We also argue that this may have implications for how occupational health in group fitness instructors is viewed, assessed and managed. Future studies may also design interventions that target key motivational factors such as autonomy (or need-) supporting environments in order to build resilience to possible eating disorders in fitness instructors. Also, the potential distinction between exercise cognitions and actual exercise behavior needs more thorough examination in future studies. Such examination should also include possibilities to examine harmonious vs. obsessive passion for exercise in exercise professionals such as group fitness instructors.

Lack of physical activity and exercise is considered a major public health challenge, and the World Health Organization aims to reduce sedentary behavior by 30% by 2030 (WHO, 2018). Exercise professionals such as group fitness instructors might therefore be considered to be important public health partners by serving as role models and motivators for increasing the amount of exercise undertaken by the general population (Thogersen-Ntoumani and Ntoumanis, 2007; De Lyon et al., 2017). Hence, the results should be used to improve fitness center managers' competence in the identification and management of such dysfunctional exercise behavior in a core occupational group of exercise professionals. The findings have relevance for the sport and exercise psychology area and the understanding of motivation and behavior in other exercising populations (i.e., athletes and recreational exercisers).

## Conclusions

To conclude, our study showed that group fitness instructors were highly intrinsically motivated for exercise, which is hypothesized to have a positive impact on their class participants. External regulation was the only motivational regulation that predicted all of the variables exercise dependence, drive for thinness, and body dissatisfaction. External regulation differences observed between instructors above and below median EDS score were independent of actual amount of exercise performed. Our findings suggest that high self-determined exercise behavior does not buffer against symptoms of exercise dependence in this special population, and thus there is a need for awareness of group fitness instructors with high scores on exercise dependence, body image concerns, amotivation, and external regulated motivation.

## Data Availability Statement

The datasets presented in this article are not readily available because of legal and ethical reasons. Requests to access the datasets should be directed to solfrid.bratland-sanda@usn.no.

## Ethics Statement

The studies involving human participants were reviewed and approved by Regional Committee for Medical Ethics in Southern Norway. The participants provided their written informed consent to participate in this study.

## Author Contributions

MR, SB-S, and JS-B contributed to conception and design of the study. SB-S organized the database. SB-S and MR performed the statistical analysis and wrote the first draft of the manuscript. JS-B wrote sections of the manuscript. All authors contributed to manuscript revision, read, and approved the submitted version.

## Conflict of Interest

The authors declare that the research was conducted in the absence of any commercial or financial relationships that could be construed as a potential conflict of interest.

## Publisher's Note

All claims expressed in this article are solely those of the authors and do not necessarily represent those of their affiliated organizations, or those of the publisher, the editors and the reviewers. Any product that may be evaluated in this article, or claim that may be made by its manufacturer, is not guaranteed or endorsed by the publisher.
